# Atypical Manifestation of LPS-Responsive Beige-Like Anchor Deficiency Syndrome as an Autoimmune Endocrine Disorder without Enteropathy and Immunodeficiency

**DOI:** 10.3389/fped.2016.00098

**Published:** 2016-09-14

**Authors:** Shahrzad Bakhtiar, Frank Ruemmele, Fabienne Charbit-Henrion, Eva Lévy, Frédéric Rieux-Laucat, Nadine Cerf-Bensussan, Peter Bader, Ulrich Paetow

**Affiliations:** ^1^Division for Pediatric Stem Cell Transplantation and Immunology, University Hospital Frankfurt, Frankfurt, Germany; ^2^UMR 1163, Laboratory of Intestinal Immunity, INSERM, Paris, France; ^3^Université Paris Descartes-Sorbonne Paris Cité and Institut Imagine, Paris, France; ^4^GENIUS Group (GENetically ImmUne mediated enteropathieS) from ESPGHAN (European Society for Pediatric Gastroenterology, Hepatology and Nutrition), Paris, France; ^5^Department of Pediatric Gastroenterology, Assistance Publique-Hôpitaux de Paris, Hôpital Necker-Enfants Malades, Paris, France; ^6^UMR 1163, Laboratory of Immunogenetics of Pediatric Autoimmune Diseases, INSERM, Paris, France; ^7^Division for Pediatric Endocrinology, University Hospital Frankfurt, Frankfurt, Germany

**Keywords:** autoimmune thyroiditis, lipopolysaccharide responsive beige-like anchor gene, autoimmune enteropathy, stem cell transplantation, genotype–phenotype correlation

## Abstract

Monogenic primary immunodeficiency syndromes can affect one or more endocrine organs by autoimmunity during childhood. Clinical manifestations include type 1 diabetes mellitus, hypothyroidism, adrenal insufficiency, and vitiligo. Lipopolysaccharide (LPS)-responsive beige-like anchor protein (LRBA) deficiency was described in 2012 as a novel primary immunodeficiency, predominantly causing immune dysregulation and early onset enteropathy. We describe the heterogeneous clinical course of LRBA deficiency in two siblings, mimicking an autoimmune polyendocrine disorder in one of them in presence of the same underlying genetic mutation. The third child of consanguineous Egyptian parents (Patient 1) presented at 6 months of age with intractable enteropathy and failure to thrive. Later on, he developed symptoms of adrenal insufficiency, autoimmune hemolytic anemia, thrombocytopenia, and infectious complications due to immunosuppressive treatment. The severe enteropathy was non-responsive to the standard treatment and led to death at the age of 22 years. His younger sister (Patient 2) presented at the age of 12 to the endocrinology department with decompensated hypothyroidism, perioral vitiligo, delayed pubertal development, and growth failure without enteropathy and immunodeficiency. Using whole exome sequencing, we identified a homozygous frameshift mutation (c.6862delT, p.Y2288MfsX29) in the *LRBA* gene in both siblings. To our knowledge, our patient (Patient 2) is the first case of LRBA deficiency described with predominant endocrine phenotype without immunodeficiency and enteropathy. LRBA deficiency should be considered as underlying disease in pediatric patients presenting with autoimmune endocrine symptoms. The same genetic mutation can manifest with a broad phenotypic spectrum without genotype–phenotype correlation. The awareness for disease symptoms among non-immunologists might be a key to early diagnosis. Further functional studies in LRBA deficiency are necessary to provide detailed information on the origin of autoimmunity in order to develop reliable predictive biomarkers for affected patients.

## Introduction

A series of monogenic primary immunodeficiency disorders (PIDs) has been described, causing a combination of immunodeficiency and multi-endocrine disorders. A prime example is the autoimmune polyendocrinopathy-candidiasis-ectodermal dystrophy syndrome (APECED) as a monogenic immune disorder caused by mutations in autoimmune regulatory (*AIRE*) gene. Further examples include immunodeficiency, polyendocrinopathy, x-linked (IPEX) syndrome (1), Stat5b-deficiency (2), CD25-deficiency (3), and CTLA-4-haploinsufficiency (4), which all can present with immunodeficiency and endocrine disorders. Mutations in Lipopolysaccharide responsive beige-like anchor (LRBA) gene have been introduced as a PID with a predominant polyautoimmune phenotype (5, 6). Affected individuals typically present with severe enteropathy in early life and may suffer from hypogammaglobulinemia, pulmonary disease, lymphoproliferative disorder (7), and infancy-onset type 1 diabetes mellitus (8). Recently, Alkhairy et al. described autoimmune thyroiditis as a part of the disease symptoms in 3 out of 31 affected patients (9). A predominant endocrine phenotype of LRBA deficiency without symptoms of enteropathy and immunodeficiency has not been described yet. Here, we describe the detailed immunology and endocrine profile of two LRBA-deficient siblings sharing the same genetic mutation with lack of LRBA protein expression. The older sibling (Patient 1) suffered from a combination of complex immune dysregulation caused by LRBA deficiency, whereas the younger sibling (Patient 2) predominantly developed multi-endocrine symptoms with thyroiditis, vitiligo, and growth retardation.

## Patients and Methods

### Patient 1

**Figure 1:**
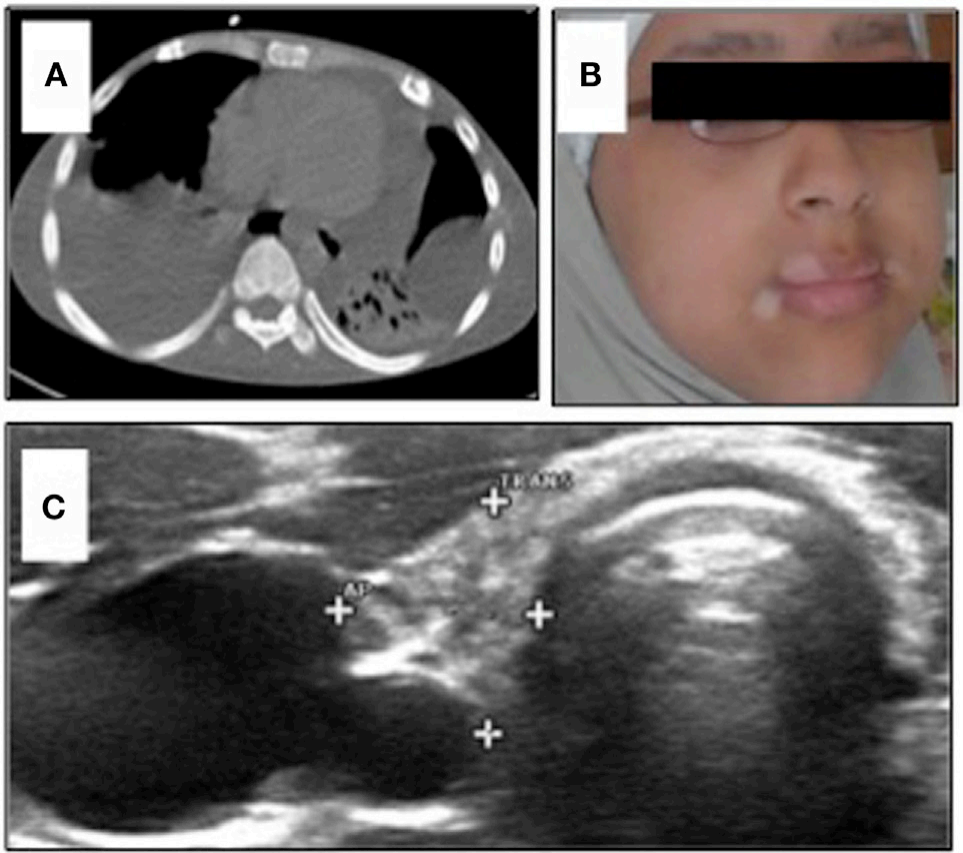
**(A)** Chest CT-scan of Patient 1 showing severe polyserositis with pleural and pericardial effusions; **(B)** patient 2 with progressive perioral vitiligo without other abnormalities in her face; and **(C)** ultrasound of the thyroid gland in Patient 2 showing a hypotrophic thyroid gland with 2.5 ml volume and several nodules <5 mm.

The third of the four children of consanguineous Egyptian parents presented, at the age of 6 months, with intractable diarrhea resulting in maldigestion and malabsorption. Over the first 4 years of life, the patient required steroid medication. Consecutively, secondary adrenal insufficiency with hypocortisolemia occurred. There was no evidence of underlying growth hormone deficiency or autoimmunity against pituitary gland and testicular tissues. Endocrine features such as growth retardation, delayed puberty, and osteoporosis were related to the severe course of chronic illness. By the age of 14 years, two episodes of autoimmune hemolytic anemia and one episode of rhabdomyolysis occurred, followed by polyserositis (Figure 1A). Initial combinatorial treatment with glucocorticoids and sirolimus resulted in short remission. However, further intensification of the immunosuppressive therapy including administration of azathioprine and tacrolimus as well as splenectomy were necessary to ameliorate autoimmunity. The severe enteropathy continued to be the predominant complaint and remained non-responsive to immunosuppressive treatment (Table 1). The functional short bowel syndrome resulted in severe cachexia with a body weight of 33.5 kg at 20 years despite long-term parenteral nutrition. The course of the disease was complicated by secondary end organ damages. The patient suffered from liver disease, esophageal varicose veins, and neuropathy. The option of allogeneic hematopoietic stem cell transplantation (alloHSCT) combined with short bowel and liver transplantation was considered at that stage. Intensified immunosuppression led to severe recurrent infections refractory to antibiotic treatment and surgical intervention. The patient lost his lower leg due to chronic osteomyelitis at the age of 21 years prior to scheduled from a HLA-identical family donor. The patient died due to recurrent septic complications and respiratory failure after lower leg amputation prior to the transplantation.

**Table 1:** **The course of the disease and clinical complications**.

	Immunosuppression
**Patient 1**	
6 months: severe pan-colitis, failure to thrive	Steroids
2 years old: chronic diarrhea, TPN-dependent	
5 years old: hearing loss	
10 years old: subclavian vein thrombosis	
14 years old: AI-hemolytic anemia, rhabdomyolysis	Splenectomy
15 years old: severe cachexia, liver failure	Ciclosporine
17 years old: recurrent GI-bleeding (ICU)	Tacrolimus
20 years old: neuropathy, seizure	Sirolimus
21 years old: 2× AI-hemolytic anemia, AI-thrombocytopenia, and polyserositis	Azathioprine
21 years old: chronic osteomyelitis (complete destruction of the joint)	
22 years old: respiratory failure, death	
**Patient 2**	
12 years old: AI-thyreoiditis, starting on l-thyroxine	None
14 years old: vitiligo	None
15 years old: 2× decompensated hypothyroidism	None

His initial laboratory results showed mild CD4^+^-lymphopenia [CD4 505/μl (NR 700–1300)] with normal distribution of CD8^+^ T-cells, NK-cells (CD3^−^CD56^+^), and B-cells (CD19^+^). IgA deficiency was observed with normal IgG, slightly reduced IgM, low tetanus vaccine antibodies, and normal hepatitis B vaccine antibodies. Thyroid hormones, growth factors, and PTH were within the normal range with low levels of vitamin D [<5 ng/ml (NR 20–30)] (Table 2). Sanger sequencing showed no *FoxP3* mutation indicative of IPEX syndrome, and the presentation was evaluated as IPEX-like.

**Table 2:** **Immunology and endocrinology work-up in two siblings with LRBA deficiency syndrome**.

Lab results	Normal range	Patient 1	Patient 2
WBC	4–11/nl	9.0	6.5
Hb	11–15 g/l	11.2	**10.7**
Thrombocytes	200–400/nl	289	185
Lymphocytes (CD3^+^)	2–4.8/nl	3.7	2.3
Granulocytes	1.8–7.1/nl	4.7	3.9
CD4^+^ T-cells	700–1400/μl	505^a^	594
CD8^+^ T-cells	200–900/μl	314^a^	585
Naive CD4^+^(CD4^+^CD45RA^+^CD62L^+^)	220–873/μl	n.a.	185
Naive CD8^+^(CD8^+^CD45RA^+^CD62L^+^)	100–470/μl	n.a.	368
DNT (CD3^+^TCRab^+^CD4^−^ CD8^−^)	0–5%	n.a.	5%
Regulatory T-cells (CD4^+^CD25^+^CD127^low^)	4–12%	n.a.	7.1%
Effector memory T4 (CD4^+^CD45RO^+^CD62L^−^)	4–20%	n.a.	9%
Central memory T4 (CD4^+^CD45RO^+^CD62L^+^)	8–50%	n.a.	48%
B-cells (CD19^+^)	100–500/μl	104^a^	164
Naive B-cells (CD19^+^CD27^−^IgD^+^)	150–515/μl	n.a.	220
Switched memory B (CD19^+^CD27^+^IgD^−^)	5–77/μl	n.a.	20
Non-switched memory B (CD19^+^CD27^+^IgD^+^)	2–77	n.a.	36
NK–cells (CD3^−^CD56^+^)	90–600/μl	95	90
IgG	590–1400 mg/dl	1159	1220
IgM	50–317 mg/dl	45	44
IgA	70–250 mg/dl	**<7**	**17**
IgE	<100 U/ml	1.2	1
Coombs test	negative	**+++^a^**	−
Anti-thrombocyte-ab	negative	−	**+**
Anti-granulocyte-ab	negative	**++^a^**	**+**
TSH	0.5–3.6 mU/l	1.7	**200**
fT4	0.9–1.6 ng/dl	1.4	**0.1**
PTH	15–65 pg/ml	24^a^	**>700**
25-OH-vitD	20–30 ng/ml	**<5**^a^	**<4**
Calcium	2.1–2.55 mmol/l	2.1	2.44
IgfBP3	2.2–4.6 μg/ml	2.1^a^	2.5
HGH	0.14–14 ng/ml	n.a.	1
IGF-1	190–805 ng/ml	466^a^	170
TG-ab	<40 IU/ml	n.a.	**>3000**
aTPO-ab	<35 IU/ml	**76^a^**	**2818**
Adrenal-ab	negative	−	−
TRAK	<1 U/l	0.03^a^	0.04
HbA1C	4.8–5.9% Hb	4.9	5.1
IFT ANA	<1:10	**1:160^a^**	**1:320**
GAD-ab	<50 mGAD/ml	n.a.	<50
Anti-IA2-Ab	<8 U/ml	<8^a^	<8
Insulin-ab	<0.4 IU/ml	<0.4^a^	<0.4
Anti-gliadin-ab	<15 U/ml	12^a^	**35**
Anti-transglutaminase-ab	<12 U/ml	10^a^	1

*Bold, abnormal values; n.a., not available*.

*^a^Values were obtained at 14 years of age*.

### Patient 2

The younger sister of Patient 1 was admitted to the endocrinology department at the age of 12 years with decompensated hypothyroidism [TSH > 200 mU/l (NR 0.5–3.6)]; fT4 0.1 ng/dl (NR 0.9–1.6), related growth retardation (<P3), pubertal arrest, and progressive perioral vitiligo (Figure 1B). Her past medical history was unremarkable with regard to relevant infections and gastrointestinal symptoms. We observed high thyroid antibody titers [aTPO-ab > 2818 IU/ml (NR < 35); TG-Ab > 3000 IU/ml (NR < 40)]. Low IGF-1 [170 ng/ml (NR 190–805)] and IGFBP3 [2.5 μg/ml (NR 2.2–4.6 μg/ml)] were detected with normal level of cortisol in plasma. Serum-calcium and phosphate were at the lowest normal range. We observed low vitamin D [<4 ng/ml (NR 20–45)], elevated AP [173 U/l (NR 47–119)], and elevated PTH [764 pg/ml (NR 15–65)]. These parameters normalized after oral calcium and vitamin D supplementation without evidence of malabsorption. Bone density – measured by peripheral quantitative CT-scan – was within the lower normal range. Ultrasound evaluation revealed a small thyroid gland [2.5 ml (NR 5.7–13.3)] with multinodular (<5 mm) texture, transformed in accordance to ongoing thyroid autoimmunity (Figure 1C). Since there was no biochemical evidence of underlying growth hormone deficiency, the progressive growth retardation in this patient was related to long-term preexisting hypothyroidism. Following initiation of medical substitution, fT4 and TSH normalized subsequently.

Echocardiography revealed a marked pericardial effusion resolving after thyroxin substitution. On further follow-up, bone age remained significantly retarded. Furthermore, slightly elevated anti-gliadin-IgG [35 U/ml (NR < 12)] was measured in presence of normal anti-transglutaminase antibodies. WBC showed mild thrombocytopenia with detectable anti-thrombocyte antibodies [185/nl (NR 200–400)] and slightly reduced hemoglobin [Hb 10.1 g/dl (NR 11–15.5)].

The lymphocyte subset analysis showed mild CD4^−^ lymphocytopenia [594/μl (NR 700–1400)] with normal naive population (CD4^+^CD45RA^+^CD62L^+^) and normal distribution of effector memory (CD4^+^CD45RO^+^CD62L^−^) and central memory (CD4^+^CD45RO^+^CD62L^+^) T-cells, normal B-cells (CD19^+^) with normal naive (CD19^+^CD27^−^IgD^+^), memory (CD19^+^CD27^+^), switched memory (CD19^+^CD27^+^IgD^−^), non-switched memory B-cells (CD19^+^CD27^+^IgD^+^), and normal NK-cells (CD3^−^CD56^+^). Tregs (CD4^+^CD25^+^CD127^low^) were within the normal range. As in her brother, IgA deficiency was detected [17 mg/dl (NR 70–300)] with normal IgG and slightly reduced IgM levels in plasma (Table 2). After thyroid hormone substitution and vitamin D supplementation, the patient re-started to grow and later experienced delayed menarche at the age of 16 years. The patient is currently 19 years old and on thyroid hormone substitution as well as oral vitamin D and calcium supplements. No immunosuppressive therapy has been initiated yet.

### Genetic Analysis Using Whole Exome Sequencing

Genetic analysis was performed after informed consent by the parents. Genomic DNA from peripheral blood cells was isolated using the QIAamp^®^ DNA Blood Mini Kit (Qiagen, Courtaboeuf, France) according to manufacturer’s instructions. Whole exome sequencing (WES) was performed on the genomic platform of Institut IMAGINE’s. Agilent SureSelect librairies were prepared from 3 μg of genomic DNA sheared with a Covaris S2 Ultrasonicator. Exons regions were captured using the Agilent Sure Select All Exon 51Mb V5 (AGILENT, Les Ulis, France) and sequenced using a HiSeq2500 next generation sequencer (Illumina) on the Genomic Platform of Institut IMAGINE, Paris. Depth of coverage obtained for each sample was around 100× with >98% of the exome covered at least 15-fold. Paired-end sequences were then mapped on the human genome reference (NCBI build37/hg19 version) using the Burrows-Wheeler Aligner. Downstream processing was carried out with the genome analysis toolkit (GATK), SAMtools, and Picard, each following documented best practices.^1^ Variant calls were made with the GATK Unified Genotyper. All variants were annotated using the in-house software (PolyWeb) developed by Paris Descartes University Bioinformatics platform. All the annotation process was based on the 72 version of ENSEMBL database. Analysis of genome variations was made using the PolyWeb software. Variants were compared to the ones already present in US National Center for Biotechnology Information database (10) of SNP, 1000 Genome, and Exome Variant Server databases. The impact on the protein function was predicted using three algorithms: Polyphen 2,^2^ SIFT (Sorting Intolerant From Tolerant, J. Craig Venter Institute), and Mutation Taster.^3^ To confirm the mutation by Sanger sequencing, genomic DNA was amplified by standard techniques using oligonucleotide primers flanking the exon 46 on the Ensembl transcrit ENST00000357115 of *LRBA* (forward 5′-TTTCCCTCCCTATTGGCAGC-3′, lower 5′-ACAGCAAGCATCTGAAGGGG-3′) using TaqDNA Polymerase (Life Technologies, Saint-Aubin, France). After purification with the QIAquick PCR Purification kit (Qiagen), PCR fragments were sequenced using the same primers by Eurofins on the Genomic Platform of Université Paris Descartes.

### Cell Culture and Immunoblotting

PBMC were collected from blood using standard density gradient separation method. Cells were either cultivated right away or frozen and activated upon thawing. T-lymphocytes were activated by staphylococcal enterotoxin E (SEE 0.1 ng/ml) or Phytohemagglutinin (PHA 12.5 μg/ml) and cultured in Panserin, 5% SAB, 1% penicillin/streptomycin, and 1% glutamine medium. At day 3 of culture and further on three times per week, IL-2 (100 ng/ml) was added to maintain cell proliferation. Cell lysates were prepared according to standard methods, separated using 3–8% Tris-acetate gels (Invitrogen), transferred onto PVDF membrane, and immunoblotted with primary antibodies to LRBA (HPA023597, Sigma), Ku70 (MA5-13110, Thermo scientific), and secondary antibodies to rabbit and mouse (Santa Cruz).

## Results

**Figure 2:**
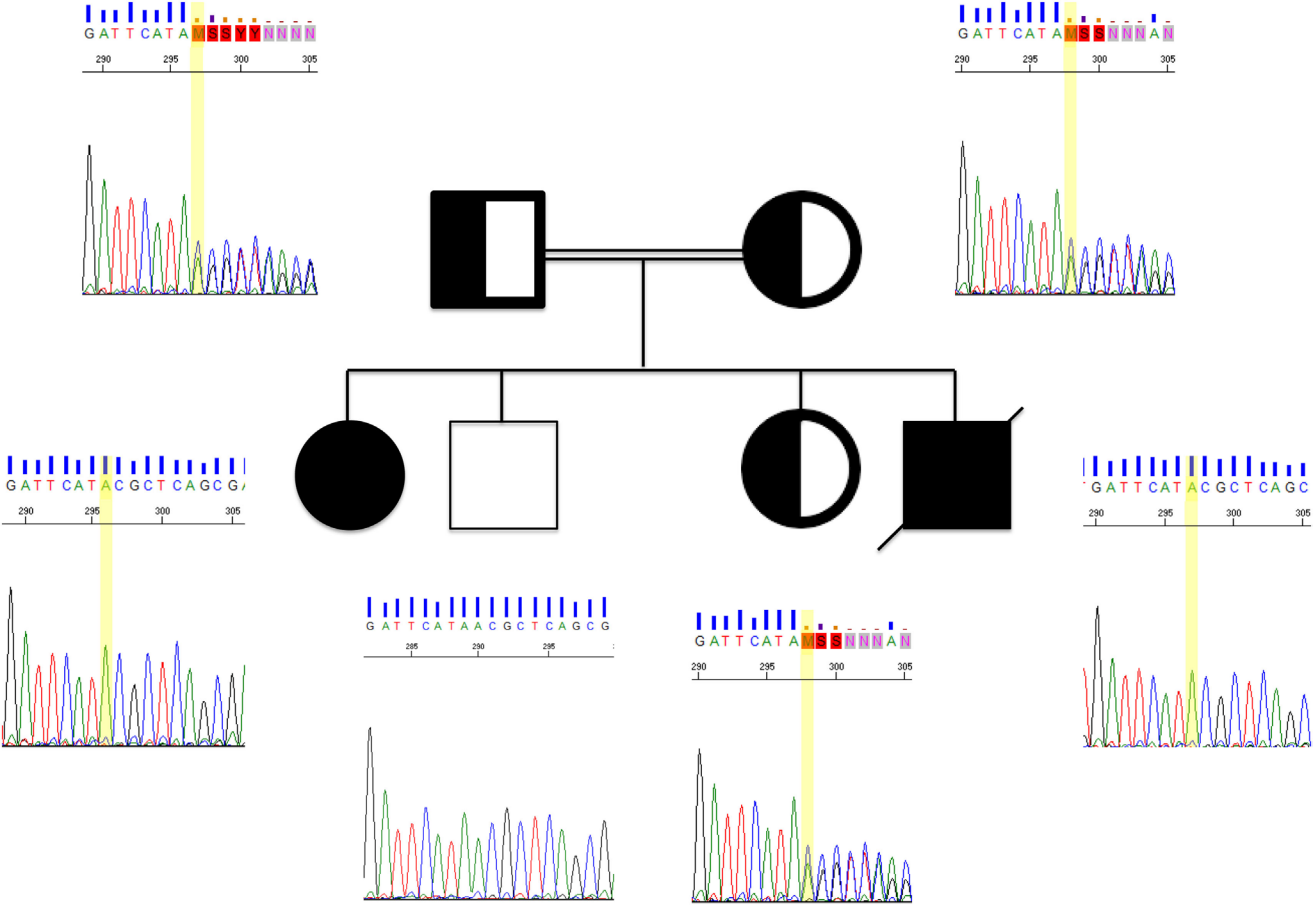
**Sanger chromatograms of two affected patients, their clinically healthy siblings, and parents**. The yellow bar is indicating a point mutation in *LRBA*. Both affected siblings found to share the same mutation, whereas their parents were heterozygous carriers. Family pedigree double lining, consanguinity; half-fill, heterozygous; solid black, homozygous.

Using WES, we identified a total of 14 genes with homozygous variants in both siblings. These homozygous variants included two genes with missense variants predicted to be damaging and one frameshift in the *LRBA* gene. The segregation of the *LRBA* variant was checked and correlated to the disease symptoms, as shown in Figure 2. None of the healthy siblings were homozygous for the *LRBA* variant. Two other homozygous variants were also detected in both affected siblings. The first variant was located in the gene encoding Fibrinogen gamma chain (*FGG* rs775086103; c.620A > G; p.Tyr207Cys). This gene has been reported in context of blood clotting disorders including familial dysfibrinogenemia, hypofibrinogenemia, and thrombophilia. There is no evidence of immunodeficiency in described patients. The second homozygous variant in both siblings was found in the gene encoding for chorionic somatomammotropin hormone 2 (*CSH2* rs549767039; c.-62A > G). This variant was located inside the 5′UTR, which belongs to the non-coding exonic sequence. *CSH2* is expressed mainly in the placenta and utilizes multiple transcription initiation sites. No association is known with immunodeficiency or autoimmunity (Table 3).

**Table 3:** **List of homozygous variants detected by whole exome sequencing in both affected patients**.

Gene	Nucleotide	Amino acid	Chromosome	Gene function
FGG	c.620A > G	p.Tyr207Cys	4q32.1	Fibrinogen gamma chain. Related to familial hypo- and dysfibrinogenemia
CSH2	c.-62A > G	Non-coding exonic	17q23.3	Chorionic somatomammotropin hormone 2, carbohydrate, and protein metabolism during pregnancy
**LRBA**	**c.6862delT**	**p.Y228MfsX29**	**4q31.1**	**B-cell function, lysosomal trafficking, and autophagy**

*Bold characters highlight the importance of the finding*.

**Figure 3:**
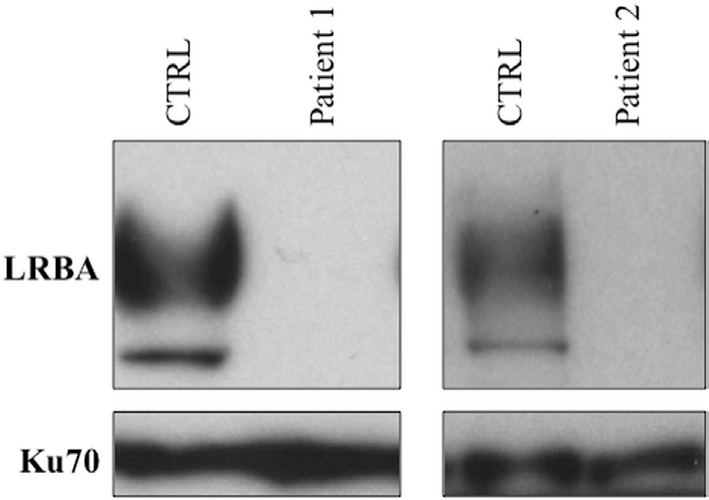
**Assessment of LRBA protein expression by Western blot**. A complete lack of LRBA protein was observed in both affected siblings.

Lipopolysaccharide responsive beige-like anchor expression was analyzed by Western blot. Compared to a healthy control, we observed a complete absence of LRBA protein in both affected siblings. The pathogenicity of the homozygous variant found by WES was confirmed by the complete lack of the LRBA protein (Figure 3).

Diagnostic work-up of the clinically healthy family members revealed an interindividual variable degree of autoimmune thyroiditis. Both LRBA-heterozygous parents showed low TSH with normal fT3 and slightly increased fT4. Thyroid antibodies (TG-ab and aTPO-ab) were detected in both parents and heterozygous sister in keeping with the diagnosis of a compensated thyroid dysfunction. The autoimmune thyroiditis in heterozygous family members is, in contrast to our Patient 2, associated with an asymptomatic clinical course of thyroid disease. Specific antibodies against adrenal tissue (21-hydroxylase) as well as antibodies typically associated with type 1 diabetes (GAD65, IA2, IAA) were proven negative in all family members. There was no history of gonadal insufficiency or impaired growth in parents or siblings. The immunology parameters were within the normal range for all of them.

## Discussion

We observed a heterogeneous clinical manifestation of LRBA deficiency in two siblings of a consanguineous family with a frameshift mutation, resulting in truncation of the LRBA protein. Our patient (Patient 2) presented with predominant endocrine symptoms of hypothyroidism, growth retardation, delayed menarche, vitiligo, and rickets but without type 1 diabetes mellitus and enteropathy or immunodeficiency.

Pediatric patients with primary immunodeficiensies may present with variable and combined multi-organ symptoms in early childhood. Heritable monogenic PID with a predominant autoimmune phenotype has overlapping symptoms with the clinical spectrum of endocrine disorders (10). Whereas some of the patients suffer from a classic combination of symptoms, others might initially and exclusively present with endocrine symptoms causing a delay in diagnosis and treatment. Early diagnosis of the underlying disease is important in order to provide optimal treatment but may be a challenge in such patients. Within the spectrum of autoimmune endocrine diseases, LRBA deficiency has to be considered in addition to IPEX, CTLA-4-haploinsufficiency, CD25^−^, and Stat5b-deficiency.

Lipopolysaccharide responsive beige-like anchor is a member of the beige and Chediak–Higashi syndrome (BEACH)-domain containing protein family. It has a significant homology with LYST, another member of BEACH family (11). LRBA is involved in critical cellular interactions such as apoptosis and autophagy and plays a fundamental role in the regulation of immune system. However, the detailed pathomechanism underlying the variable symptom complex in LRBA deficiency is not completely understood yet.

Maintaining the balance between immunity and autoimmunity requires a complex interaction of activating and inhibiting factors. Firstly described in 1987, the glycoprotein CTLA-4 (12), mainly localized in intracellular vesicles of Tregs, is a key regulator molecule in this cascade (13). By acting as an early checkpoint, CTLA-4 has a major influence on maintaining self-tolerance (14, 15). There is evidence that LRBA controls CTLA-4 expression and thereby supports its transendocytotic function by preventing its degradation in recycling lysosomes (16). In support, Tregs of LRBA-deficient patients expressed lower intracellular and membrane levels of CTLA-4 without an alteration of the CTLA-4 mRNA. Two LRBA-deficient patients with detectable moderate residual LRBA protein were reported to have higher CTLA-4 levels suggesting a quantitative correlation between CTLA-4 deficiency and residual LRBA protein in LRBA-deficient patients (16).

The recent description of the extended phenotype of the disease in 22 genetically confirmed cases of LRBA deficiency showed immune dysregulation (95%), organomegaly (86%), recurrent infections (71%), and hypogammaglobulinemia (57%) as the main clinical complications, whereas 81% of these LRBA-deficient patients had normal T-cell counts, and 73% had reduced Tregs numbers (6). Both patients reported by Schreiner et al. suffered from endocrine symptoms in addition to underyling enteropathy and immunodeficiency (8). Autoimmune thyroiditis was observed by Alkhairy et al. in 3 out of 31 patients with underlying LRBA deficiency syndrome (9). However, none of these patients presented with an autoimmune thyroiditis as the major clinical manifestation. GH deficiency *per se* has not been associated with LRBA deficiency. Indeed, most of the affected patients seem to develop secondary growth failure in addition to their gastrointestinal dysregulation and malabsorption. This is a common feature of a series of other primary immunodeficies as well. However, Patient 2 did not suffer from gastrointestinal disease or malabsorption previously. There was no biochemical evidence of underlying growth hormone deficiency. The progressive growth retardation in this patient was related to long-term preexisting hypothyroidism. As observed in this cohort and in additional reports, the clinical course of the disease remains highly variable (6, 9).

The suggested selective regulation of CTLA-4 degradation by LRBA is tempting to compare immune defects of LRBA with CTLA-4-deficient patients. Schubert et al. reported 19 patients with a genetically confirmed CTLA-4-haploisufficiency (4). Only 12 patients presented with severe clinical manifestations including enteropathy (78%), hypogammaglobulinemia (76%), granuloma (66%), autoimmune thrombocytopenia (35%), and autoimmune hemolytic anemia (28%). Autoimmune thyroiditis was present in two patients as a part of a polyautoimmune disorder. Isolated autoimmune endocrine disorder was not reported in this cohort (4). Recently, Slatter et al. reported the outcome in a group of CTLA-4 deficient patients (*n* = 8) undergoing alloHSCT. Autoimmune endocrine disorders (type 1 diabetes, exocrine pancreas insufficiency and thyroiditis) were reported in two patients as one part of their complex autoimmune disorder. Interestingly, signs of isolated endocrine autoimmunity were observed among family members of these two patients suggesting a minor disease activity in these individuals without being diagnosed as CTLA-4-deficient previously (17).

As mentioned above, the majority of LRBA- and CTLA-4-deficient patients show a relevant Treg dysfunction with immune dysregulation as their main clinical symptom resulting in an IPEX-like disorder. However, a series of proven CTLA-4-deficient individuals are asymptomatic despite disturbed Treg cell suppressive ability (4). As observed in our Patient 2, LRBA deficiency can also present with normal T- and B-cell subpopulations and Treg numbers. Whether there is a correlation between the disease severity and the degree of Treg dysfunction in LRBA-deficient individuals needs to be analyzed in larger cohorts prior to initiation of immunosuppressive treatment. Furthermore, the lysosomal sorting function of LRBA might not be limited to T-lymphocytes and to CTLA-4, opening possibilities to evaluate symtptoms of these patients, which are not fully explained by a Treg-restricted dysfunction. To what extent additional modifiers such as epigenetic or environmental factors might influence the disease outcome in LRBA- and CTLA-4-deficient patients, remains to be analyzed in further studies.

Since first description in 2012, about 50 LRBA-deficient patients have been detected and the number is increasing. Very recently Lévy et al. described two patients suffering from arthritis as their main clinical symptoms (18). We believe that the awareness of the heterogeneity of this disease among non-immunologists might be the key to the early detection and initiation of the treatment of LRBA deficiency. Without appropriate treatment, there is a high mortality in a majority of the affected patients. While some of the patients die early due to enteropathy and complications of the immunosuppressive treatment, others remain stable on systemic immunosuppression with glucocorticoids as single agents or as part of combinatorial therapy (6). Due to the lack of genotype–phenotype correlation in this disease and the clinical heterogeneity, the underlying diagnosis might remain undetected in some patients and lead to delayed treatment. These patients need early multidisciplinary care involving specialists in the fields of endocrinology, gastroenterology, and immunology. Supportive medical therapy of the evolving endocrine insufficiencies is indicated at an early stage with continuous follow-up to detect further organ manifestations. Some patients may remain refractory to standard therapy. Extended immunosuppressive therapy may be necessary for patients with progressive symptoms of the disease and might result in stable disease but also can lead to lethal complications. Recently abatacept, a CTLA-4 fusion protein, was shown to provide improvement in LRBA-deficient patients (16). There is no long-term evaluation of efficacy and safety of the treatment available yet. In patients suffering from threatening autoimmunity, enteropathy and infectious complications of the immunosuppressive treatment early stem cell transplantation could be the only curative treatment option. A few LRBA-deficient patients being treated successfully by stem cell transplantation have been reported (6, 19). Further studies on transplantation in LRBA deficiency are necessary to evaluate the optimal conditioning regimen and transplantation-related problems such as immune reconstitution posttransplantation and the risk of graft versus host disease. Given the severe fatal course of the disease in Patient 1, the establishment of a long-term treatment strategy including evaluation of a preemptive alloHSCT seems crucial for Patient 2 at this stage.

In light of a quite limited genotype–phenotype correlation, further data on LRBA-deficient patients are necessary to establish predictive prognostic biomarkers. Furthermore, detailed data on LRBA-deficient patients with an endocrine phenotype are necessary to evaluate the long-term risk of endocrine disorder, their outcome, and the optimal early treatment options in these patients.

## Ethics Statement

This study was performed with parental permission and approval by the ethic committee of University Hospital Frankfurt.

## Author Contributions

All authors contributed to the conception and interpretation of data. SB, UP, and PB provided clinical data and wrote the manuscript. EL, FC-H, FR-L, FR, and NC-B provided data on functional testing and genetics.

## Conflict of Interest Statement

The authors declare that the research was conducted in the absence of any commercial or financial relationships that could be construed as a potential conflict of interest.

## Footnotes

^1^http://www.broadinstitute.org/gatk/guide/topic?name=best-practices

^2^http://genetics.bwh.harvard.edu/pph2/

^3^www.mutationtaster.org

## Abbreviations

Ab, antibodies; aTPO, antithyroperoxidase; AP, alkaline phosphatase; BEACH, the beige and Chediak–Higashi syndrome-domain; GAD65, glutamat-decarboxylase; IGFBP3, insulin-like growth factor-binding protein 3; IA2, islet autoantibody-2; IAA, insulin autoantibodies; IGF, insulin-like growth factor; LYST, lysosomal trafficking regulator; LPS, lipopolysaccharide; PHA, phytohemagglutinin; PTH, parathormone; SEE, staphylococcal enterotoxin E; TSH, thyroid-stimulating hormone; TG, thyroglobulin.
